# “The great escape”: how an incident of elopement gave rise to trauma informed palliative care for a patient experiencing multiple disadvantage

**DOI:** 10.1186/s12904-024-01374-x

**Published:** 2024-02-28

**Authors:** Sam Quinn, Libby Ferguson, Derek Read, Naomi Richards

**Affiliations:** 1 End of Life Studies Group, University of Glasgow, Dumfries Campus, Dumfries and Galloway, Scotland, DG1 4ZL UK; 2 Marie Curie Scotland, 133 Balornock Road, Stobhill, Glasgow, Scotland, G21 3US UK; 3 Glasgow, Scotland UK

**Keywords:** Palliative care, Trauma informed care, Post-traumatic stress disorder, Poverty, Homelessness, Veteran, Elopement, Patient autonomy, Flexibility in care, End of life care

## Abstract

**Background:**

This case report from Scotland, UK illustrates how unresolved traumatic experiences across the life course can affect a patient’s engagement with palliative care and offers insights into the flexibility and adaptations necessary for taking a trauma informed approach to care for an individual experiencing multiple disadvantage. Trauma informed care is a cornerstone in the pursuit of equitable palliative care, particularly for those facing multiple disadvantage, as it acknowledges the impact of past traumas on current healthcare experiences, and fosters an environment of understanding, acceptance, and tailored support to alleviate suffering in the final stages of life.

**Case presentation:**

“M” was a veteran with a history of homelessness, living with end stage anal cancer and symptoms consistent with post-traumatic stress disorder, although he never received a formal diagnosis. M exhibited complex behaviours perceived to be related to his history of trauma, including his decision to elope from the hospice, reluctance to accept personal care from nurses, and unpredictability. These behaviours posed a significant challenge to his palliative care team, both in the hospice and at home. An individualised and flexible approach to care delivery was eventually adopted, which included a ‘safety-netting’ approach and care delivery outside of the hospice. M was ultimately supported to remain at home until a week before he died.

**Conclusion:**

M’s case underscores the necessity of adopting a trauma informed approach to palliative care, particularly for patients with a history of trauma and multiple disadvantage. The case highlights the importance of understanding and respecting a patient’s past traumas, promoting safety and autonomy, and ensuring flexibility in care delivery.

## Background

Empathy means realising no trauma has discrete edges. Trauma bleeds. Out of wounds and across boundaries [1].

Trauma refers to experiences or situations that are emotionally painful and distressing that overwhelm an individual’s capacity to cope. It is not limited to physical injury but can result from severe psychological stress [2]. Trauma can result from a single event, or from prolonged, repetitive stress such as experiencing poverty [3, 4]. Individuals who face multiple and intersecting disadvantage are at higher risk of trauma and associated mental health issues [3]. Multiple disadvantage refers to a range of intersecting social, economic, and health-related factors that can negatively affect an individual’s access to, experience with, and outcomes from healthcare [5]. For example, factors such as experiences of ‘deep and persistent’ poverty across the life course, homelessness/insecure housing, and substance use complicate the management of care in healthcare systems that have been critiqued for overrepresenting the interests of the white middle class [6, 7].

Trauma informed care has been recognised internationally as a key approach to reducing health inequities and enhancing the well-being of those most at risk [2, 8, 9]. It is a strengths-based framework that acknowledges the extensive impact of trauma, which can accumulate across the life course, and its potential to substantially influence the experiences of individuals seeking healthcare. The goal of this approach is to prevent re-traumatisation and to support resilience and recovery. Central to this is a shift in perspective, moving from asking “What’s wrong with you?” to exploring “What happened to you?“ [10].

While trauma informed care gained prominence in the early 1990s [11], its application to palliative care is relatively new [12]. Post-traumatic stress disorder (PTSD), as defined in the DSM-V, is a disorder where individuals experience persistent re-experiencing, avoidance, negative thoughts and feelings, and heightened arousal following exposure to traumatic events [13]. The prevalence rate of PTSD in the general population is estimated to be around 8%, although it is substantially higher for certain groups such as people living in more deprived neighbourhoods, yet there remains a lack of routine screening for trauma related symptoms in the palliative care population [14]. To support the development of trauma informed care in the palliative care specialism, there is an urgent need for examples of good practice and skills demonstrated by trauma informed palliative care teams [15].

To contribute to this knowledge base, we present the case of ‘M’, a 65-year-old male diagnosed with cancer and living with experience of ‘deep, persistent’ poverty [16], and prior experience of homelessness following his time in the army. M was a participant in our longitudinal study Dying in the Margins (https://www.gla.ac.uk/research/az/endoflifestudies/projects/dyinginthemargins/), when two months before his death, he made a self-proclaimed ‘great escape’, eloping from the hospice where he was receiving care for a recurrent tumour in his anal canal, precipitating the mobilisation of a police helicopter and an on-the-ground search team, including police dogs.

Unsurprisingly, this development caused turmoil in the hospice and made us as researchers question whether M’s care had been sufficiently trauma informed. To understand M’s elopement from the hospice, and whether his care was sufficiently trauma informed, we present an account of M’s case compiled from three data sources: (1) a first-hand written account provided by M’s friend and long-term carer, ‘D’; (2) a first-hand account written by M’s palliative medicine consultant and; (3) visual and textual data collected during a larger research study, Dying in the Margins; investigating experiences of poverty and deprivation at the end of life, in which M was a participant from May 2021-September 2021.

Data were analysed using thematic analysis [17], which involved systematically coding the written accounts provided by co-authors #2 and #3, alongside the visual data taken by M himself and a professional photographer, and identifying patterns, themes, and insights to enhance our understanding of trauma informed palliative care in the context of multiple disadvantage. The design of this research aligns with current literature advocating for more participatory research in palliative and end of life research [18].

## Ethical statement

All methods were performed in accordance with relevant guidelines and regulations. All experimental protocols were approved by a named institutional/licencing committee. Specifically, participatory visual methods and interview data collection were approved by the North of Scotland NHS Research Ethics Committee (REC) on 4th April 2020 (Reference: 20/NS/0032). Informed consent was obtained from all participants to publish identifying information and images in an online open-access publication.

## Case presentation

### Friend and long-term carer’s perspective

M was a big man, with a big heart and a big personality. He used humour in all aspects of his life and had a tremendous sense of fun. But he was also very complicated. M wanted everything right and done his way, which probably harked back to his army training. He served for nearly 13 years in the Royal Highland Fusiliers. In one year, he had lost six ex-army friends through drink or suicide.

When M undertook his great escape from the hospice, I think he planned it as a military exercise. He looked at the weakness of the security and got out of the ward. Once he was home in the flat, [another friend] and I were his main carers. He wouldn’t accept help from [outside] carers in terms of personal care.

I think sometimes he was in denial about the trauma he had experienced. He always said he didn’t want counselling. He had a psychologist at one point, who diagnosed him with PTSD, but he was very dismissive. I think some of his behaviours support the diagnosis. He would take the dog out to the park at night because he couldn’t sleep, he would use humour to cope, and sometimes our relationship could be very volatile.

**Fig. 1:**
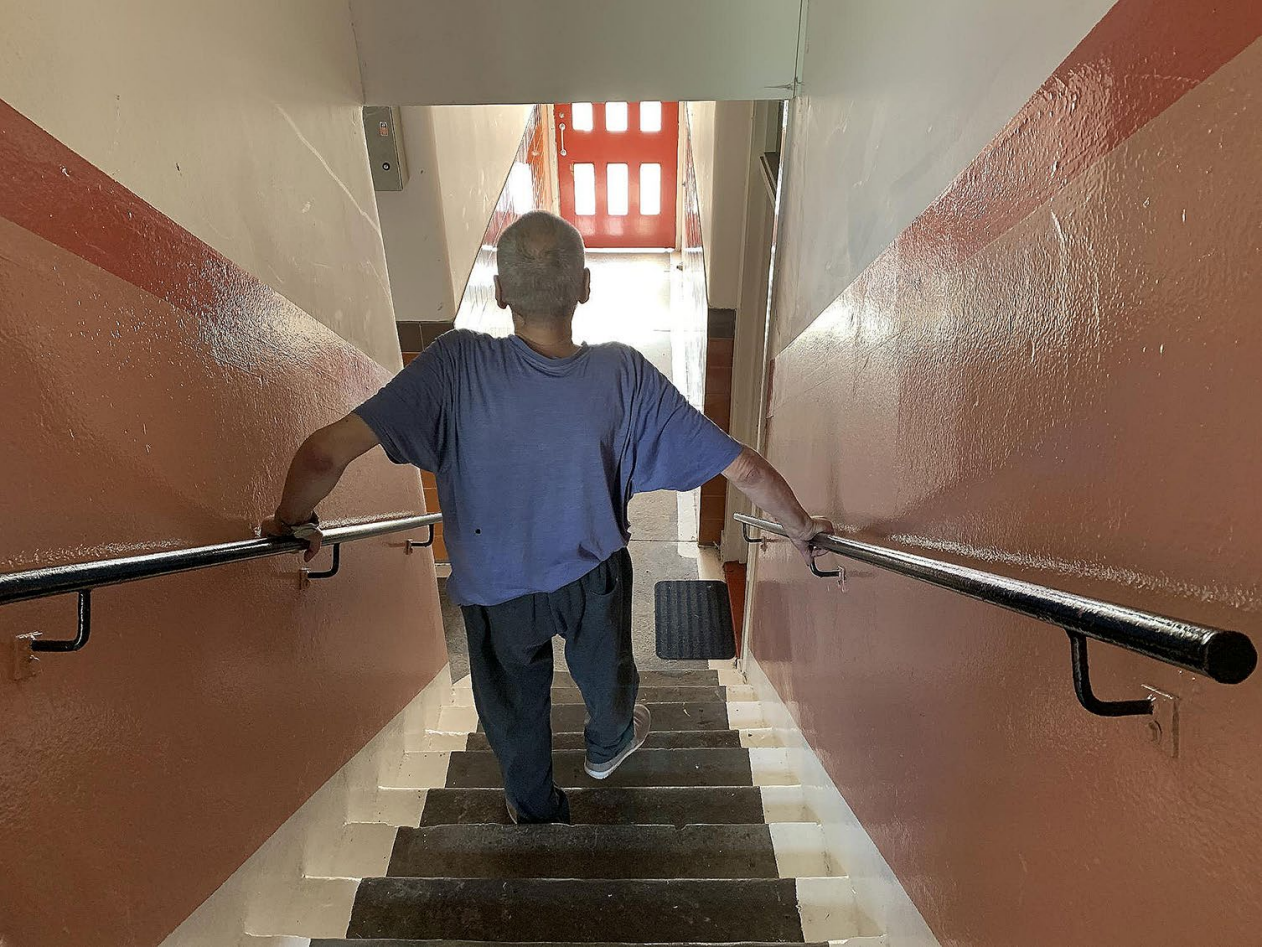
“M” on the stairs to his flat (Image © Margaret Mitchell, CC BY-NC)

Being M’s carer towards the end was knackering, partly because of the unpredictability of his personality, partly because of his condition and partly because of where he lived. His flat was on the second floor of a block of flats, and it was difficult for him to get up and down the stairs (See Fig. 1).

A week before he died, he was in a lot of pain and there were problems with his wound, so he asked the consultant to help him get into the hospice. After a few days, M was more stable, and I could focus on spending time with him at the end.

### Palliative medicine consultant’s perspective

When I arrived at the hospice on the morning of the great escape, it was not a complete surprise that M had left during the night. He was an independent, humorous character who preferred to cope by himself. Although not formally diagnosed, he described post-traumatic stress disorder following time in the army. He had experienced homelessness for several years on his return and often spoke of this, alongside regrets around involvement in gangs and violence in his youth.

He had a diagnosis of recurrent squamous cell carcinoma of his anal canal for which he had declined salvage surgery. He was initially referred to the Palliative Medicine clinic for symptom control. The tumour was fungating and caused severe neuropathic anal pain ‘like electric shocks’ which made it difficult for him to sit. A fistula with the base of his penis meant he was constantly damp with malodourous discharge, causing him distress and embarrassment. He had been seen at home and in the clinic before an earlier hospice admission where symptoms improved with titration of opioids, adjuvants, and antibiotics. Pain control remained challenging, and we discussed referral for consideration of an intrathecal device. M felt this would be ‘too intense’ and was clear he did not wish for additional hospital appointments or procedures.

His sudden departure had been triggered by a request from the hospice nurse to lock several hundred pounds in cash in the bedroom safe. He had been readmitted with delirium related to infection. Whilst this gradually improved with antibiotics, he remained paranoid at times. He began to find the hospice environment restrictive and decided to leave. The nurses tried to persuade him to stay but recognised that this worsened his agitation.

He was able to walk short distances but was limited by weakness and significant pain which required regular analgesia. That morning he left without his walking frame, house keys and medication. I was worried he would be in severe pain as analgesia wore off, possibly stuck at the side of the road somewhere. We contacted the police and explained that whilst M had capacity and was not detainable, we were concerned for his wellbeing. The police searched the local area with dogs and a helicopter. Somehow M managed to travel across the city to a friend’s flat. The friend called the hospice to let us know he was safe.

**Fig. 2:**
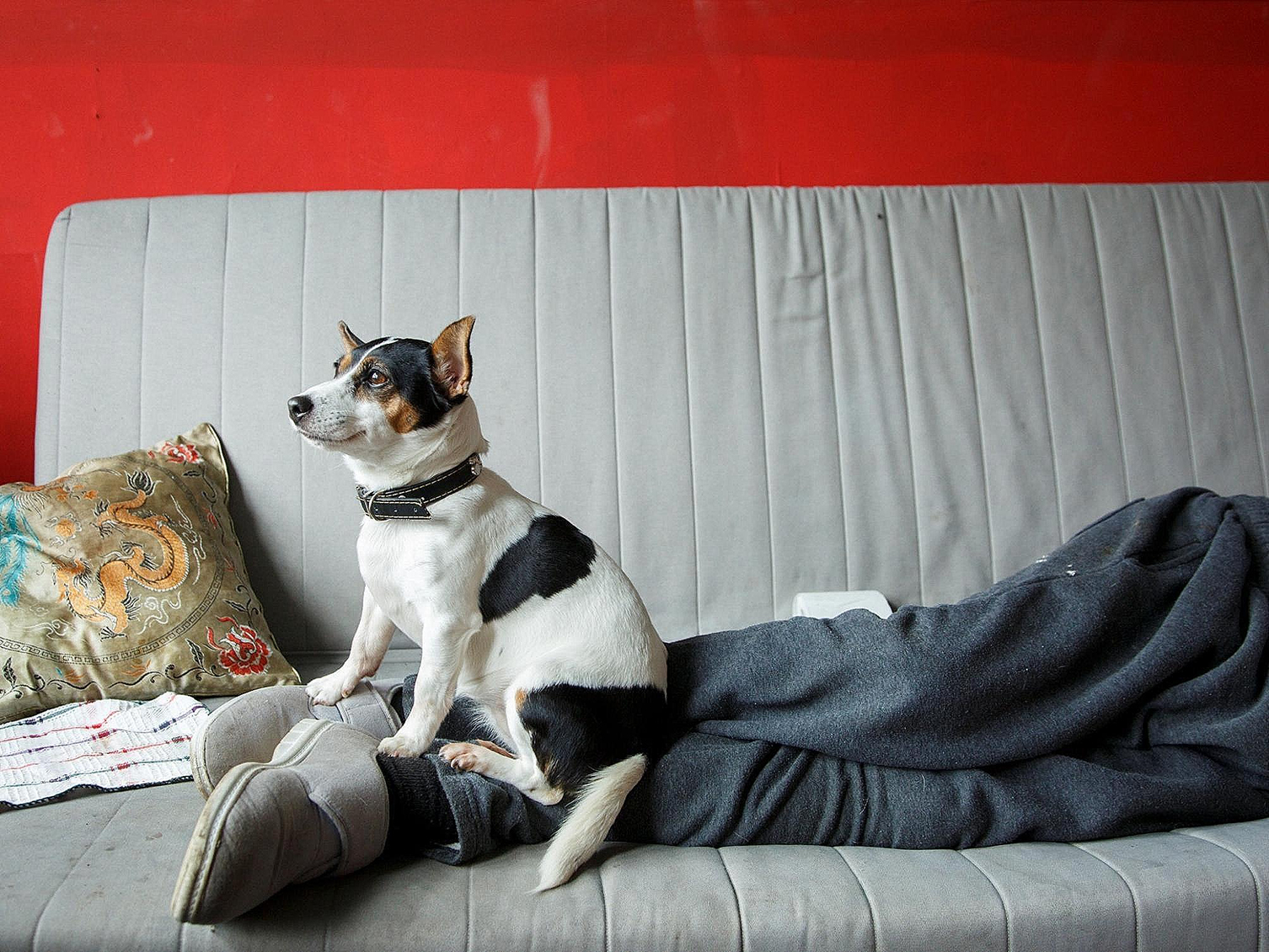
Lily the dog on M’s legs (Image © Margaret Mitchell, CC BY-NC)

I knew M was likely to require inpatient care in the future, so it was important to maintain his trust in the team. I had previously undertaken training in trauma informed practice through NHS Education for Scotland. M did not want to come back into the hospice building but agreed to catch up in the car park. He hugged me and laughed; we walked around the car park talking things over. M wished to remain at home with his Jack Russell, Lily (see Fig. 2). To try and respect M’s autonomy and choice we were flexible around follow-up with a safety-netting approach, although there were limits on the help he would accept.

We recognised that M’s engagement with the hospice was likely to fluctuate. M’s flat was often busy with unannounced visitors sometimes drinking, sometimes using drugs. We initially organised support with hospice nurses following discharge, but he was often out at the pub when they called round. M would often miss palliative care outpatient appointments but phone for advice instead.

### Discussion and conclusions

Thematic analysis of M’s case revealed several key themes including independence and autonomy, financial security and control, trauma and PTSD, rigidity in the healthcare system, social relationships and community, identity and self-perception, suitability of the home and living environment, and caregiver experience and burden.

Analysing what we learned about M’s case, we argue that his behaviour was shaped by undiagnosed and untreated trauma from earlier in his life. Notably, a psychologist suggested to M that he was likely to have PTSD, leading M to disengage from mental health services. Both veterans and people with lived experience of homelessness are at increased risk of post-traumatic stress disorder [19], which can lead to a higher likelihood of hospitalisation in the final 12 months of life and increased symptom burden, including a higher risk of experiencing delirium at the end of life [20]. Despite these risks, these patients can be reluctant to engage with healthcare institutions that symbolise inflexible and oppressive systems of institutional control [7, 21]. The UK Adult Psychiatric Morbidity Survey indicated that up to 70% of people with PTSD are not receiving any form of treatment. Given the scale of undiagnosed and untreated trauma, it is essential to recognise that the absence of a formal PTSD diagnosis should not prevent clinicians from implementing a trauma informed approach, particularly when patients have complex histories suggestive of traumatic experiences.

Responding to M’s elopement, the hospice eventually adopted a trauma informed approach to his care [14]. However, opportunities to avert his elopement may have been missed. For example, a trauma informed approach might interpret M’s reluctance to leave his money in a safe as a response rooted in his past experiences of homelessness, and the need to maintain control over his personal belongings for a sense of security. Instead of requiring M to put his cash in the safe, a money belt could have been provided for him to keep the cash on his person. M’s experience demonstrates how the decisions made by hospice staff, such as attempting to secure his cash and involving the police, could inadvertently criminalise patients who are already grappling with financial hardship and multiple disadvantage [22]. It is important to recognise that M was within his rights to retain his money and to leave the hospice. Such decisions may exacerbate the feelings of mistrust and discomfort that individuals experiencing multiple disadvantage might already have toward healthcare institutions and their staff.

The challenges faced by M in receiving appropriate care during his stay at the hospice are not unique, as many individuals with multiple disadvantage encounter systemic barriers in receiving healthcare that relies on traditional modes of delivery. The experience of stigma, exclusion, and power imbalances can result in those requiring palliative care being reluctant to engage with medical institutions. Unfortunately, such mistrust of healthcare professionals can be amplified by a lack of culturally safe and trauma informed care [18]. In this case, a ‘safety netting’ approach to consultation [23] was eventually adopted, which appeared to work. Safety netting refers to the process of providing patients or their carers with information about actions to take if their condition fails to improve, or changes, or if they have further concerns about their health in the future, acting as a contingency plan to manage uncertainty.

In providing trauma informed care, the team had to accept that while M may not be at home for scheduled visits or attend outpatient appointments when arranged, this approach was needed to maintain M’s trust. A retrospective cohort study using general practice data from Scotland (*n* = 824,374) over 3 years found missed appointments to be a significant risk factor for all-cause mortality, particularly in patients with mental health conditions. Patients who are viewed as not engaging or declining input may appear to relieve pressure on the system, but this ‘missingness’ can inadvertently increase health inequalities and potentially increase the overall burden of morbidity [24]. Meeting the needs of patients who regularly miss appointments requires a level of flexibility not always present in existing healthcare appointment systems. However, fostering equitable palliative care means adopting a flexible approach to individual needs and offering care outside of traditional hospice and hospital settings [21].

While examples of trauma informed practice in palliative care are slowly increasing [25], there is still significant scope for innovation and education [14]. Recognising that patients receiving palliative care are likely to have a history of trauma, we call for work to embed trauma informed practice into palliative care and stress the value of example cases and shared learning to achieve this. Strategies for delivering trauma informed palliative care arising from this case and our review of existing evidence are presented in Table 1.

**Table 1:** Strategies for delivering trauma-informed palliative care

Strategy	Description
Recognition of Undiagnosed Trauma	Healthcare practitioners, within a multidisciplinary team, should not rely solely on formal diagnoses of PTSD or other trauma-related conditions. Familiarity with signs of undiagnosed trauma and symptoms consistent with PTSD is crucial.
Fostering Trusting and Honest Relationships	Focus on developing trust through clear, honest communication and a non-judgmental approach. Encourage active listening, empathy, and validation of patient experiences to create a safe, respectful space for open dialogue.
Sensitivity to Patient Autonomy and Belongings	It is important to respect the significance of personal belongings and autonomy, particularly for patients with histories of homelessness or unstable living conditions. Handling personal belongings with sensitivity can improve a patient’s sense of security and autonomy.
Flexible Approach to Care Delivery	Adopting a non-traditional approach in palliative care delivery, accommodating the unique needs of each patient. This includes flexibility in scheduling and understanding that adherence to conventional appointment schedules may not be feasible for all patients.
Safety Netting Approach	Implementing a contingency plan in consultations by providing patients or their carers with actionable information for scenarios where the patient’s condition might worsen or if they have further health concerns.
Addressing Power Imbalances and Stigma	Awareness of potential power imbalances, stigma, and exclusion within healthcare settings is essential. Actions that may exacerbate feelings of mistrust and discomfort in healthcare institutions should be avoided.
Education and Training in Trauma-Informed Care	Training for healthcare providers on the link between trauma and multiple disadvantages across the life course is vital. Understanding the cyclical nature of trauma and socio-economic disadvantage can help in addressing the needs of such patients more effectively.
Recognising ‘Missingness’ as a Risk Marker	Not recognising patients who miss appointments or seem disengaged can inadvertently increase health inequalities. It is important to develop strategies to effectively engage these patients.
Adopting Innovation in Care Delivery	Emphasising the need for flexibility, responsiveness, and innovation in care delivery. Tailoring care to meet the unique needs and circumstances of each patient is key to fostering equitable palliative care.

In conclusion, addressing the needs of individuals experiencing multiple disadvantage is essential for providing equitable palliative care as it ensures that all individuals, regardless of their socioeconomic status, personal circumstances, or other disadvantage, have access to quality end of life care. Adopting a trauma informed approach can help practitioners foster trust with patients who are experiencing multiple disadvantage. However, delivering trauma informed care involves moving beyond traditional models and embracing flexibility, responsiveness, and innovation in care delivery. Education and sharing learning on trauma informed palliative care can help healthcare professionals better support patients facing complex challenges, ultimately enhancing the quality of end of life care provision.

Key Implications:

- A formal diagnosis should not be a prerequisite for implementing trauma informed care. Practitioners should familiarise themselves with signs of undiagnosed trauma and symptoms consistent with post-traumatic stress disorder (PTSD).
- Patients with a history of trauma, poverty, and other disadvantage may be hesitant to engage with medical institutions. It is crucial to recognise the potential for power imbalances, stigma, and exclusion within healthcare settings, and to avoid actions that may exacerbate these feelings of mistrust and discomfort. Given “M"’s resistance to having his money kept in a safe, we recommend sensitivity towards how personal belongings are handled, especially for patients with histories of homelessness or unstable living conditions. Practitioners should consider that for some, retaining control over personal belongings might be intrinsically tied to an individual’s sense of security and autonomy.
- ‘Missingness’ is a significant risk marker, particularly for patients with mental health issues. Trauma informed palliative care means moving beyond traditional models of palliative care delivery and offering services that are flexible to individual circumstances and where more time and effort are expended on developing conditions of safety and trust. Recognising that a conventional appointment-based model may not always be suitable for patients with histories of trauma, we recommend offering more flexible scheduling options. This could include allowing patients to choose appointment times or offering drop-in services.
- People experiencing multiple disadvantage have an increased likelihood of having experienced traumatic events and are at an elevated risk of mental health issues. Practitioners should receive training on the link between trauma and multiple disadvantage experienced across the life course, often beginning in childhood. This training should emphasise that trauma can lead to, or exacerbate, social and economic disadvantage, which can, in turn, increase the risk of further traumatic experiences. Understanding this cycle can better equip healthcare providers to address the needs of patients who have faced such intersecting challenges.

## Data Availability

The datasets generated and/or analysed during the current study are not publicly available due to being unable to anonymise data and the specificity of the study.

## Abbreviations

NHS: National Health Service
PTSD: Post-Traumatic Stress Disorder
TIC: Trauma Informed Care
